# Validation of the Intermolecular Disulfide Bond in Caspase-2

**DOI:** 10.3390/biology13010049

**Published:** 2024-01-17

**Authors:** Megan E. Amason, Lupeng Li, Carissa K. Harvest, Carolyn A. Lacey, Edward A. Miao

**Affiliations:** 1 Department of Integrative Immunobiology, Duke University School of Medicine, Durham, NC 27710, USA; 2 Department of Molecular Genetics and Microbiology, Duke University School of Medicine, Durham, NC 27710, USA; 3 Department of Microbiology and Immunology, University of North Carolina at Chapel Hill, Chapel Hill, NC 27599, USA; 4 Department of Pathology, Duke University School of Medicine, Durham, NC 27710, USA; 5 Department of Cell Biology, Duke University School of Medicine, Durham, NC 27710, USA

**Keywords:** caspase-2, apoptosis, disulfide bonds, oxidative damage, ferroptosis

## Abstract

**Simple Summary:**

Caspase-2 belongs to a family of proteins that are involved in cell death pathways. Studies involving caspase-2 have not led to a clear verdict on either the upstream activating signal or the downstream substrates of activated caspase-2. Here, we investigate the unique and highly conserved ability of caspase-2 to form a disulfide-bonded dimer. Disulfide bonds can serve structural roles, but they can also alter the enzymatic activity and localization of proteins within the cytosol. We show that caspase-2 must first dimerize for the disulfide bond to form. This finding suggests that disulfide bond formation is a regulated mechanism of activation. We also investigated various stimuli, some that have been previously published in the context of caspase-2 and some that have not, in caspase-2-deficient cells. Our results show partial protection in caspase-2-deficient cells for some of the stimuli, but a true activating signal, where the loss of caspase-2 is completely protective, has yet to be found.

**Abstract:**

Caspases are a family of proteins involved in cell death. Although several caspase members have been well characterized, caspase-2 remains enigmatic. Caspase-2 has been implicated in several phenotypes, but there has been no consensus in the field about its upstream activating signals or its downstream protein targets. In addition, the unique ability of caspase-2 to form a disulfide-bonded dimer has not been studied in depth. Herein, we investigate the disulfide bond in the context of inducible dimerization, showing that disulfide bond formation is dimerization dependent. We also explore and review several stimuli published in the caspase-2 field, test ferroptosis-inducing stimuli, and study in vivo infection models. We hypothesize that the disulfide bond will ultimately prove to be essential for the evolved function of caspase-2. Proving this will require the discovery of cell death phenotypes where caspase-2 is definitively essential.

## 1. Introduction

Caspases, or *c*ysteine–*asp*artic prote*ases*, were first identified in the 1980s for their function in cell death during embryonic development [1,2,3]. It is now appreciated that caspases are involved in many heterogenous contexts, including the immune response to intracellular infection [4,5,6]. Broadly, caspases are divided based on their function in apoptosis as initiators and executioners, or in facilitating pyroptosis [7]. Diverse signals will activate specific caspases, which then cleave specific protein substrates. All caspases use a catalytic cysteine residue to cleave after an aspartic acid residue, meaning the mechanism of catalysis is well conserved [8].

Caspases must undergo dimerization and/or proteolytic cleavage to become activated. Many of the caspase family members have an N-terminal *ca*spase *r*ecruitment *d*omain (CARD) that facilitates activation via homotypic interactions with upstream proteins. For example, the CARD of APAF-1 can oligomerize with the CARD of caspase-9, resulting in activation that culminates in apoptosis [9]. This high-molecular weight platform is known as the apoptosome. Analogously, it is thought that PIDD1 senses a cellular perturbation and then recruits RAIDD to form the PIDDosome, allowing the CARD of RAIDD to bind the CARD of caspase-2 [10,11]. However, the requirement of PIDD1 and RAIDD during caspase-2 activation remains controversial [12,13]. Regardless, it is more widely agreed that dimerization of caspase-2 is essential for its activation. Based on this requirement and its sequence homology to caspase-9, caspase-2 has been classified as an initiator caspase, which is further supported by the observation that the overexpression of caspase-2 causes apoptosis.

Despite this, the published phenotypes for caspase-2 are contradictory in that caspase-2 sometimes causes cell death, but in other cases causes cell cycle arrest [14,15]. Cell cycle arrest would be a curious function for a caspase family member, as other well-characterized caspases result in cell death. A contributing factor to the dissensus in the field is that different investigators use diverse cell types, and the responses appear to be cell-type specific. Another major limitation of the literature is the inconsistent use of assays, readouts, and validation strategies between studies [16]. Of the numerous caspase-2 phenotypes that have been published, there are a few that have gained popularity: caspase-2 may be involved in the response to DNA damage, the protection against microtubule disruption, the suppression of tumorigenesis, and the response to endoplasmic reticulum (ER) stress. However, few of the published phenotypes have been validated using knockout cells or mice. Interestingly, *Casp2*^−/−^ mice are overtly normal [17], which is in contrast to the embryonic lethality associated with other initiator caspase knockout mice (*Casp8* and *Casp9*) [18,19]. Although it was the second caspase to be identified [20], the collection of caspase-2 studies in the field has not led to a clear verdict on either the upstream activating signal or its downstream substrates.

One intriguing aspect of caspase-2 was revealed in the crystal structure of the dimerized protein: an intermolecular disulfide bond forms at the geometric center of the dimer, covalently linking the monomers together [21]. This is a unique feature of caspase-2 that appears to stabilize the dimer in solution. A follow-up study mutated the cysteine residue involved in the disulfide bond (C436) [22]. When the investigators compared wildtype (WT) caspase-2 to the C436G disulfide bond mutant, they observed a decrease in, but not an elimination of, the catalytic activity of purified protein. They also observed a decrease in, but again not an elimination of, its ability to induce apoptosis in an overexpression system. However, because these were intermediate phenotypes and the disulfide bond was not required for dimerization of caspase-2, no further studies have taken place to investigate the role of the disulfide bond in caspase-2.

Many proteins that are destined for secretion contain disulfide bonds that are acquired during folding in the ER [23]. Intermolecular or intramolecular disulfide bonds stabilize these secreted proteins, serving an important structural role [24]. In contrast, it is unusual for proteins in the cytosol to contain disulfide bonds because the cytosol is a highly reducing environment [25]. Therefore, it is an intriguing observation that caspase-2 is capable of forming a disulfide-bonded dimer because caspase-2 and its proposed activating platform, the PIDDosome, are located in the cytosol [26]. However, cysteine residues in the cytosol can be sensitive to oxidation-reduction (redox) changes, and examples of specific proteins that use cysteine modifications as a post-translational control mechanism have been elucidated [24,27,28,29].

Redox homeostasis, defined as the balance between reducing and oxidizing reactions, plays an essential role in many cellular processes, including differentiation, development, metabolism, and even the immune response to infection [30]. Key sites and processes where potentially harmful oxidants, referred to as reactive oxygen species (ROS) or reactive nitrogen species (RNS), are generated include the mitochondria during ATP synthesis, the ER during protein folding, and the cellular membrane during the immune response [31,32]. Of critical importance for maintaining redox homeostasis, despite the generation of these harmful byproducts, is glutathione (GSH), the most abundant antioxidant in cells [33,34]. The ratio of reduced glutathione (GSH) to the oxidized form (GSSG, also known as glutathione disulfide) is an important buffering system in the cell because GSH can spontaneously reduce oxidants. The ability of GSH to cycle between its reduced and oxidized forms also makes it an essential cofactor in enzymatically controlled antioxidant responses [35]. The intracellular GSH pool is tightly monitored and regulated due to its importance during a myriad of cellular processes. Importantly, all three of the aforementioned species (ROS, RNS, and GSH) have been identified as secondary messengers capable of reacting with free thiol groups (SH) to facilitate the formation of disulfide bonds within or between proteins [27,36].

We hypothesize that the ability of caspase-2 to form a disulfide bond, which is unique and highly conserved, plays a critical role in caspase-2 activation in vivo. Herein, we sought to determine whether the disulfide bond is required for cell death phenotypes associated with caspase-2.

## 2. Materials and Methods

### 2.1. Crystal Structure Analysis

The crystal structure of dimerized caspase-2 (NCBI PDB ID 1PYO) was visualized using the NCBI (Bethesda, MD, USA) iCn3D Structure Viewer.

### 2.2. Molecular Cloning

To generate the various caspase-2 constructs, the caspase-2 sequence (NM_032982.4) was inserted into the dimerization vector pHom-1 (previously known as pC_4_-F_v_1E), which contains the DmrB domain (previously known as Fv) using the SpeI cut site and standard cloning procedures [37]. The HA tag was subsequently switched to a FLAG tag, using IVA cloning [38]. IVA cloning was also used to perform site-directed mutagenesis of various residues, as indicated. Finally, DmrB–Casp2–FLAG constructs were inserted into the pMXs-IP vector, using PacI and NotI restriction enzymes and standard cloning procedures. The final constructs were confirmed using Sanger sequencing.

### 2.3. A549 Cell Culture

To generate A549 cells that expressed our various caspase-2 constructs, Phoenix amphotropic cells were used to generate retroviral particles, as previously described [39,40]. On the morning of day zero, Phoenix cells were seeded into a 6-well tissue culture-treated plate and transfected in the evening using Lipofectamine 3000 (according to the kit protocol), containing either a control pMXs-IP–eGFP plasmid (for assessing transfection and transduction efficiency) or a pMXs-IP–DmrB–Casp2–FLAG plasmid containing each caspase-2 construct. On day three, 2 mL of cDMEM (for the Phoenix and A549 cells: 4.5 g/L D-Glucose DMEM supplemented with 10% heat-inactivated FBS and 1X PenStrep) was added to each well. On day four, A549 cells were seeded into a 6-well tissue culture-treated plate at a density of 0.75 × 10^5^ cells/well. On day five, the supernatant from the Phoenix cells was harvested, filtered through a 0.45 µm syringe filter, and polybrene was added at a working concentration of 5 µg/mL, before being added to the A549 cells. The plates containing the A549 cells and the viral supernatant were spun at 2000× *g* for 2 h at room temperature, before being returned to the incubator. After one hour, the supernatant was aspirated and replaced with cDMEM. The GFP expression and transduction efficiency were monitored over time using an Invitrogen™ (Carlsbad, CA, USA) EVOS™ inverted fluorescent microscope, and the transduced A549 cells were selected with 1.0 µg/mL puromycin for three passages, or until the mock transduced cells died. The transduced cells were expanded and maintained below passage 15.

### 2.4. Inducible Dimerization, Generation of Lysates, Treatment of Lysates

To induce dimerization, transduced A549 cells were seeded at 2 × 10^5^ cells/well in 6-well, tissue culture-treated plates, and treated the following day. The cell supernatant was replaced with cDMEM containing 5 nM of AP20187, unless otherwise indicated. To harvest, media was collected and the wells were rinsed with PBS before adding 0.25% Trypsin–EDTA to lift the cells. The collected cells were spun at 450× *g* for 5 min, at room temperature. The cell pellets were resuspended in approximately 50 µL lysis buffer (1% IGEPAL in PBS) and lysed on ice for 5 min. The lysates were then spun at 21,000× *g* for 10 min at 4 °C. The supernatants were then collected for BCA protein concentration analysis, according to the kit protocol. In some cases, the lysates were further treated with the indicated dose of SNAP or NONOate for 1 h, or 2 mM DSS crosslinker for 30 min.

### 2.5. SDS–PAGE and Immunoblotting

For Western blot analysis of the cell lysates, BCA standardized samples were combined with Laemmli sample buffer (with or without the addition of βME), boiled at 95 °C for 5 min, and loaded into 8–16% or 4–15% polyacrylamide gels. The gels were run at 100 volts for approximately 1 h and 30 min. The transfer of the protein onto membranes was performed at 100 volts for 1 h, followed by blocking in 5% milk/TBST for 1 h. The membranes were gently rocked overnight at 4 °C in the primary antibody diluted in 5% BSA/TBST (rat anti-Caspase-2 at 1:2000) (mouse anti-FLAG at 1:5000). The following day, the membranes were gently rocked for 1 h at room temperature in the secondary antibody diluted in 5% milk/TBST (goat anti-rat at 1:10,000) (goat anti-mouse at 1:10,000), before visualization via ELC using an Azure Biosystems (Dublin, CA, USA) Azure 500^®^ infrared fluorescent imager. The images were further processed using Fiji by ImageJ, version 1.54g.

### 2.6. Genotyping

To genotype C57BL/6 (referred to as WT; from Jackson Laboratories (Bar Harbor, ME, USA)) and *Casp2*^−/−^ mice [17], the Kapa genotyping kit was used, according to the kit protocol. The cDNA from ear punches was used as a PCR template and amplified, according to the Jax protocol.

### 2.7. Primary Cell Isolation and Culture

BMMs were generated as previously described [41]. On day zero, tibias and femurs were harvested from 8–10-week-old, age- and sex-matched C57BL/6 WT, or *Casp2*^−/−^ mice, before moving the bones into a biosafety cabinet. The ends of the bones were cut off to expose the marrow, and marrow was flushed into a Petri dish with cold cDMEM (for BMMs: 4.5 g/L D-Glucose DMEM supplemented with 10% heat-inactivated FBS, 15% L-cell conditioned medium, and 1X Pen/Strep). Marrow was pooled within each genotype and within each sex, filtered through a 70 µm strainer, before being centrifuged at 300× *g* for 10 min at 4 °C. The resulting cell pellets were resuspended in DMEM and transferred to five, 15 cm non-treated dishes containing 20 mL cDMEM, or eight, 10 cm non-treated dishes containing 10 mL cDMEM. On day three, an additional 10–20 mL of cDMEM was added to each dish. On day seven, the cells were lifted with PBS–EDTA (1 mM) and either frozen in freezing media (10% DMSO in FBS) using a Mr. Frosty™ freezing container or seeded for experiments.

### 2.8. Cell Treatments and Assessment of Apoptosis via Annexin-V and PI

The cells were seeded in 24-well plates (non-treated for BMMs) at a density of 0.5 × 10^5^ cells/mL/well. The next day, the media was aspirated and replaced with 1 mL cDMEM containing each stimulus. The amount of DMSO (*v*/*v*) did not exceed 0.5%. For the heat shock treatment of cells, the plates containing cells cultured at 37 °C were placed into an incubator warmed to 43 °C for 30 min or 60 min. In some experiments, the media was first replaced with cDMEM that was pre-warmed to 43 °C. The cells were then returned to a 37 °C incubator to recover for 24 h. To harvest the cells at the indicated times, the supernatant and cells were collected in flow tubes using PBS–EDTA (for BMMs) and spun at 300× *g* for 5 min at room temperature. The supernatant was decanted and the cells were stained for Annexin-V and propidium iodide (PI), according to the kit protocol. Importantly, the cells were stained with Annexin-V for 15 min (protected from light), followed by the addition of PI and immediate analysis using a BD LSRFortessa™ X-20 cell analyzer. The final analysis was performed using FlowJo™ (for Windows, version 10.7.1).

### 2.9. Preparation of Inoculum

The bacteria were grown overnight on brain heart infusion (BHI) agar plates (*C. violaceum* and *L. monocytogenes*) or chocolate agar plates (*F. philomiragia*) at 37 °C and stored at room temperature for no more than two weeks. To prepare the infectious inocula, the bacteria were cultured in 3 mL BHI broth with aeration overnight at 37 °C, before being diluted in PBS to the indicated inoculum.

### 2.10. In Vivo Infections

All the mice were housed according to the IACUC guidelines at UNC-Chapel Hill or at Duke University, with approved protocols in place. For the in vivo infections, 8–10-week-old, age-matched mice were infected as previously described [42]. The mice were infected intraperitoneally (*C. violaceum* and *F. philomiragia*) or intravenously (*L. monocytogenes*) with the indicated number of bacteria in 200 µL PBS. Whole livers and spleens were harvested at the indicated timepoints.

### 2.11. Plating for CFUs

At the indicated days post-infection (DPI), the mice were euthanized, and the spleen and liver were harvested using aseptic technique, as previously described [42]. Briefly, the spleens were placed in a 2 mL homogenizer tube with 1 large metal bead and 1 mL sterile PBS, and the whole livers were placed in a 7 mL homogenizer tube with 1 large metal bead and 3 mL sterile PBS. The tube weights were recorded before and after tissue harvest to normalize the CFUs/volume/tissue. After homogenizing, 1:5 serial dilutions were performed in sterile PBS, and the dilutions were plated on BHI (*C. violaceum* and *L. monocytogenes*) or chocolate agar (*F. philomiragia*) in triplicate or quadruplicate. The following day, the bacterial colonies were counted and the CFU burdens calculated.

### 2.12. Statistics

Statistical analysis was performed using GraphPad Prism 9.5.1. The data were first assessed for normality using the Shapiro–Wilk test. For two groups, a two-tailed *t*-test (or Mann–Whitney test for abnormally distributed data) was used. For the experiments with multiple factors, a two-way ANOVA was used.

## 3. Results

### 3.1. Inducible Dimerization of Caspase-2

**Figure 1:**
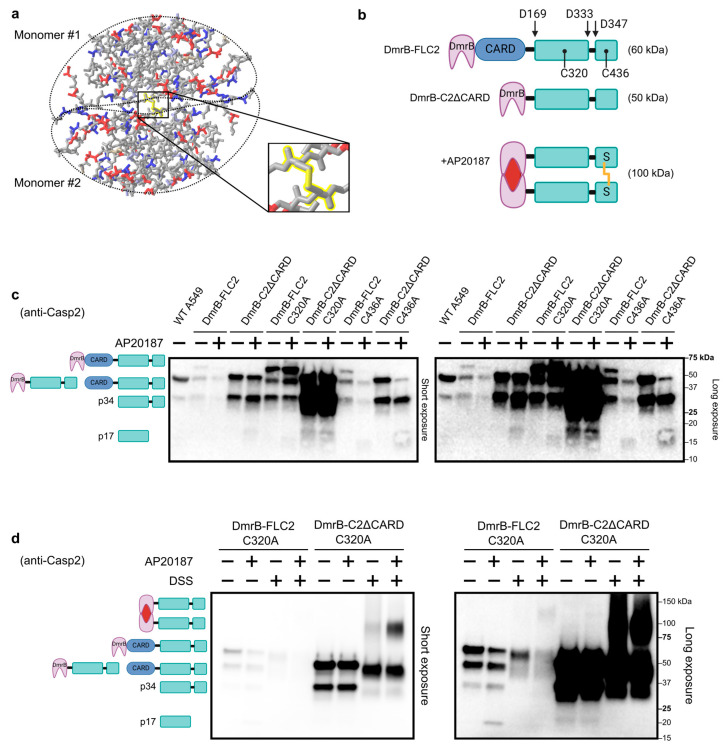
Caspase-2 inducible dimerization system. (**a**) Crystal structure of dimerized caspase-2 published in [21]. The structure is colored by charge: positive (blue), partial-positive (light blue), negative (red), and neutral (grey). Disulfide bond highlighted in yellow. (**b**) Schematic of various caspase-2 constructs with N-terminal FKBP (DmrB) tag, with indicated residues of autoproteolytic cleavage (D169, D333, and D347), catalytic cysteine (C320), and cysteine involved in disulfide bond (C436). Addition of AP20187 causes dimerization. Disulfide bond formation is depicted with yellow line between thiol (SH) groups in small subunits. (**c**) Western blot analysis of whole cell lysates from transduced A549 cells, stably expressing various caspase-2 constructs. Prior to lysis, cells were treated with DMSO (control) or AP20187 for 4 h. (**d**) Western blot analysis of whole cell lysates as in (**c**), but cells were treated with DMSO or AP20187 for 2 h. Lysates were then treated with PBS (control) or DSS crosslinker for 30 min.

Based on the location of the disulfide bond within the crystal structure (Figure 1a and Figure S1a–c), we hypothesized that dimerization was required to bring the two cysteines close enough for the disulfide bond to form. To test this, we used the iDimerize™ Inducible Homodimer System to tag caspase-2 with an FKBP domain [26,43], which can be dimerized through the addition of a small molecule called AP20187. We generated several N-terminally tagged caspase-2 constructs (Figure 1b). We added the dimerizer domain (DmrB) as an N-terminal fusion to full-length caspase-2 (DmrB-FLC2) or used DmrB to replace the N-terminal CARD (DmrB-C2ΔCARD). We then used retroviral transduction to generate stably expressing cell lines. We chose the A549 human adenocarcinoma cell line because previous reports showed caspase-2 activation in these cells [44], and A549 cells have high transduction efficiency [45]. Although A549 cells have endogenous caspase-2, we reasoned that these cells would allow us to study dimerization of our various caspase-2 constructs in a biologically relevant cell type.

The full-length caspase-2 construct (FL-C2) was expressed at relatively low levels (Figure 1c, uncropped blot in Figure S5), presumably because autoactivation resulted in the loss of the high-expressing cell population. This is in agreement with prior studies showing that the overexpression of caspase-2 causes apoptosis [20]. Interestingly, the construct without the CARD (C2ΔCARD) expressed at higher levels than the full-length construct, suggesting that autoactivation occurs, at least in part, through CARD–CARD interactions. For both FL-C2 and C2ΔCARD, the introduction of a catalytic cysteine mutation (C320A) caused much higher expression levels. Thus, the C2ΔCARD C320A construct expressed at the highest levels (Figure 1c). Furthermore, the mutation of the disulfide bond cysteine (C436A) in both FL-C2 and C2ΔCARD did not enhance expression to the same extent as seen for the catalytic cysteine mutation (C320A). This is in agreement with the previous observation that the disulfide bond is dispensable for the dimerization and activation of caspase-2 [22]. This supports a model where the disulfide bond is dispensable for caspase-2 activation if driven solely by overexpression.

Cleavage of caspases has long been used as a readout for activation [8]. Indeed, cleavage of caspase-2 has been used by many groups as a surrogate for its activation; although, it is worth mentioning that different groups report different cleavage fragments to denote active caspase-2 (i.e., p34, p17, and p12) [16]. An intriguing observation is that various caspase-2 cleavage products sometimes exist in unstimulated cells [44,46,47]. In untransduced and untreated WT A549 cells, we consistently observed a cleavage product around 34 kDa, which is likely generated through autoproteolytic cleavage of endogenous caspase-2 at residue D169 (Figure 1b,c). The cleavage product (p34) was also present for each of our transduced constructs, including the C320A catalytic mutant, which is presumably cleaved by endogenous caspase-2. When the small molecule AP20187 was added to induce dimerization, both the FL band and the p34 cleavage product were diminished for FL-C2 and for the C436A mutant constructs (Figure 1c), and a faint band at approximately 17 kDa was also visible in some cases. Although the p17 cleavage fragment was generated as a result of inducible dimerization, the majority of cells appeared healthy during microscopic inspection (Figure S2), suggesting that dimerization alone is not sufficient to cause apoptosis in these cells. It is a curious observation that inducible dimerization of caspase-2 does not cause cell death in the majority of cells, because using the same inducible dimerization system with caspase-9 has been well-documented to cause apoptosis [48].

To confirm that we could induce the dimerization of our constructs, we treated cell lysates with DSS crosslinker to maintain the protein–protein interactions. We chose cells expressing the catalytic cysteine mutant for the following reasons: (1) these constructs were expressed at higher levels and allowed for easier detection, and (2) because they are unable to undergo autoproteolytic cleavage, dimerized constructs likely persist for a longer window of time, which also increases sensitivity for detection. The addition of AP20187 again led to the p17 cleavage product in cells that expressed the FL-C2 C320A construct (Figure 1d, long exposure), indicating that inducible dimerization of our mutant construct activated endogenous caspase-2. The C2ΔCARD C320A construct, which expressed at the highest levels, showed low levels of dimerization without AP20187, which was enhanced when AP20187 was added to the cells prior to lysis. Interestingly, the p34 cleavage product that is visible with or without AP20187 was not visible in lysates treated with DSS (Figure 1d); this can be explained if the p34 fragment is loosely associated with the CARD (which is not bound by the antibody), and when these two are crosslinked, they become the full-length size. We also performed dose curve and time course experiments to assess the effectiveness of AP20187 in dimerizing our constructs. Dimerization can be observed with as low as 5 nM of AP20187 and within 30 min (Figure S1d).

### 3.2. Disulfide Bond Formation in Dimerized Caspase-2

**Figure 2:**
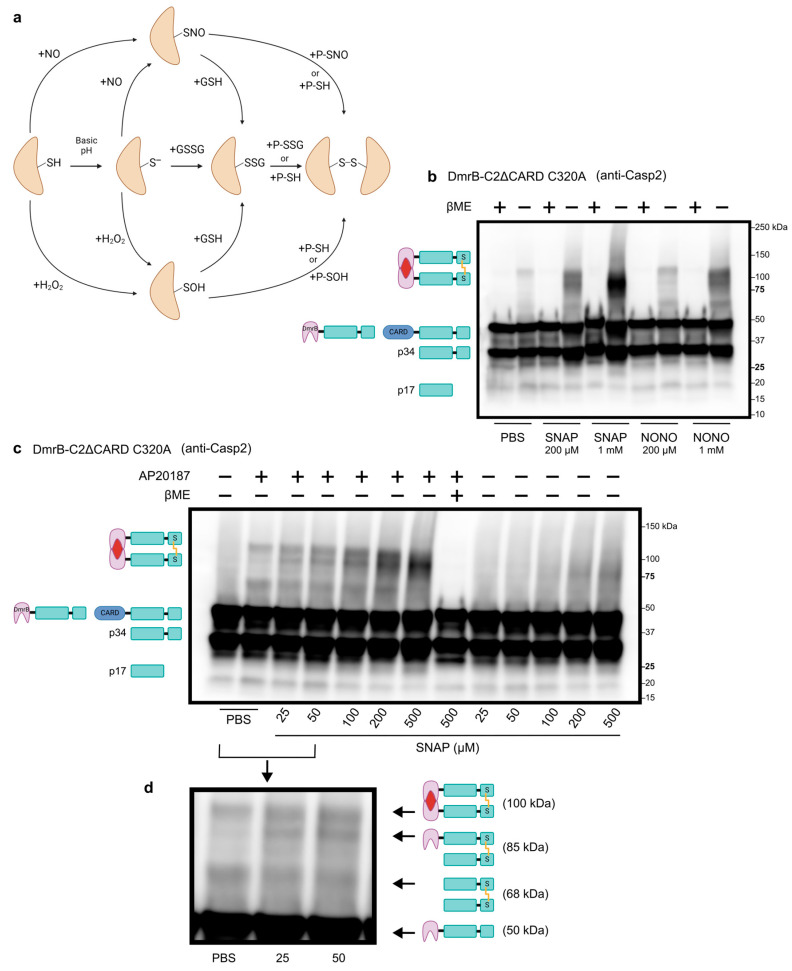
Caspase-2 disulfide bond formation is dimerization dependent. (**a**) Schematic of oxidation reactions that can induce disulfide bond formation. Abbreviations: thiol (SH), nitric oxide (NO), S-nitrosylated protein (SNO), S-sulfenylated protein (SOH), thiolate (S^−^), glutathione (GSH), glutathione disulfide (GSSG), S-glutathionylated protein (SSG). (**b**) Western blot analysis of whole cell lysates from transduced A549 cells stably expressing DmrB-C2ΔCARD C320A. Prior to lysis, cells were treated with AP20187 for 4 h. Lysates were then treated with PBS (control), SNAP, or NONOate for 1 h. (**c**) Western blot analysis of whole cell lysates as in (**b**), but cells were treated with DMSO (control) or AP20187 for 2 h. Lysates were then treated with PBS or SNAP for 1 h. (**d**) Magnification of lanes 2–4 showing three distinct bands, corresponding to the molecular weight of various cleavage products.

After confirming that we could induce dimerization of caspase-2, we wanted to assess the ability of dimerized caspase-2 to form the disulfide bond. Disulfide bonds usually form through oxidation reactions. Although reactive oxygen species such as hydrogen peroxide can facilitate these reactions, nitric oxide (NO) has a high specificity for reacting with thiol groups to form S-nitrosylated proteins [49]. Indeed, recent evidence suggests that S-nitrosylated proteins are relatively unstable, transient intermediates, and disulfide bonds are the more favorable end products [49] (Figure 2a). To test whether NO could induce disulfide bond formation in dimerized caspase-2, we treated lysates with nitric oxide donors SNAP or NONOate. Simply excluding the reducing agent βME from the sample buffer allowed us to see that a small amount of disulfide-bonded caspase-2 forms in lysates treated with PBS (Figure 2b, uncropped blots in Figure S6). The formation of the disulfide bond was greatly enhanced when lysates were treated with SNAP or NONOate, in a dose-dependent manner (Figure 2b). Importantly, including βME in the sample buffer successfully reduced the disulfide bond and abolished the 100 kDa bands. Furthermore, disulfide bonds did not form in the absence of AP20187 (Figure 2c), demonstrating that dimerization is required for disulfide bond formation. In addition to the expected band at 100 kDa, we also noticed two other distinct bands for lysates treated with 25, 50, or 100 µM SNAP (Figure 2d). These bands correspond with disulfide bond formation of various cleavage products in which one or both monomers have been cleaved at D169.

The ability of caspase-2 to form a disulfide-bonded dimer is unique, and that dimerization is first required suggests a regulated mechanism of control. Taken together, we hypothesize that caspase-2 has a two-step activation mechanism: (1) dimerization, and (2) disulfide bond formation. This also explains why dimerization alone is not sufficient to cause apoptosis in the majority of cells.

### 3.3. Experimental Strategy to Study Cell Death Phenotypes in Caspase-2-Deficient Cells

With the in vitro dimerization assay, we showed that dimerization is required for disulfide bond formation. However, using transduction in cell lines has several major caveats. First, endogenous caspase-2 is present and is activated by the overexpression of our constructs. Second, transduction results in higher caspase-2 expression than is physiologically correct. Third, immortalized cells are well adapted to oxidizing environments due to their growth in atmospheric oxygen. We therefore wanted to interrogate the requirement of the disulfide bond in a cell type in which caspase-2 could be manipulated under physiologically correct expression levels. We first wished to use primary cells from knockout mice to find a phenotype where the loss of caspase-2 was protective and, subsequently, we could test the role of the disulfide bond in that phenotype by generating disulfide bond mutant C436A mice. Not all published stimuli have been tested in knockout cells, although the most extensively studied caspase-2 knockout cells are immortalized mouse embryonic fibroblasts (MEFs). We chose to work in bone marrow-derived macrophages (BMMs) for the following reasons: (1) It has been reported that caspase-2 is inhibited during mitosis through phosphorylation [50]. If caspase-2 is mitotically regulated, immortalized MEFs may spend a greater amount of time with caspase-2 phosphorylated and inhibited due to their increased proliferative state. Although BMMs proliferate after isolation and during differentiation, their rate of replication is slower compared to MEFs, and they have a finite number of cell divisions before they enter senescence and die. (2) BMMs are an easily obtained source of primary cells that allow the interrogation of various genotypes. (3) Many caspase family members have immune-related phenotypes. BMMs do express caspase-2 [51], but the role of caspase-2 in these immune cells has not been extensively explored. Finally, (4) few studies have used BMMs, and we wished to advance the field by testing various stimuli in this cell type. Therefore, we chose to search for a caspase-2-dependent cellular phenotype in BMMs, reasoning that the discovery of a phenotype would allow us to test the requirement of the disulfide bond.

**Figure 3:**
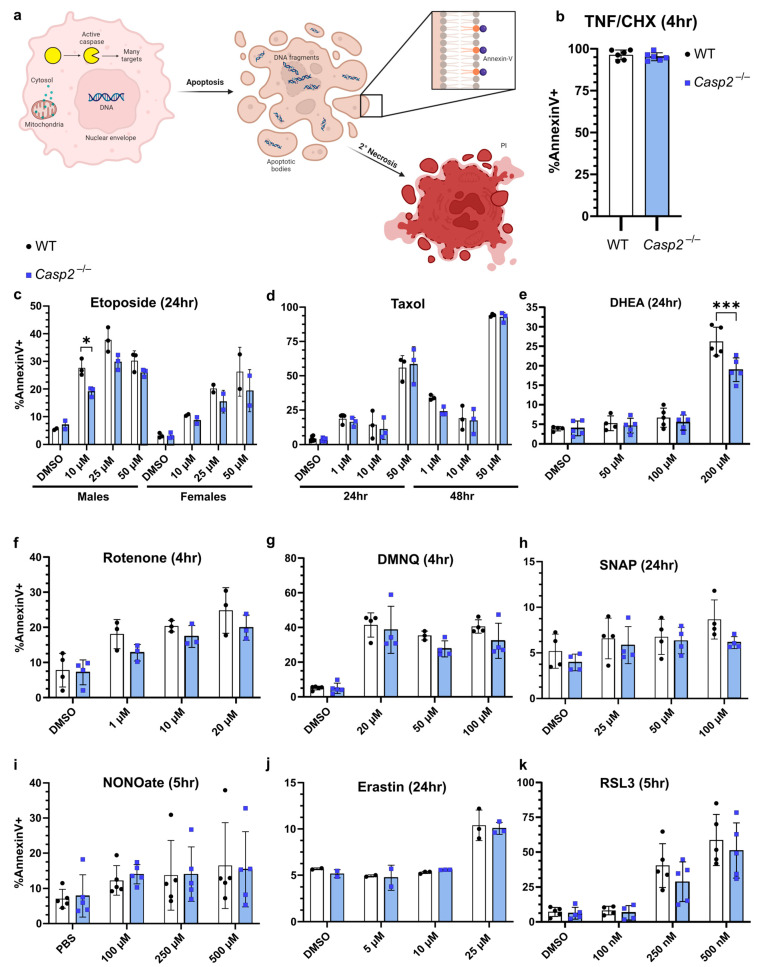
Investigation of cell death phenotypes in *Casp2*^−/−^ BMMs. (**a**) Schematic of measurable events during apoptotic cell death used by various studies (mitochondrial permeability, cytochrome c release into the cytosol, caspase activation via cleavage or enzymatic activity, DNA fragmentation, cell shrinkage, phosphatidyl serine exposure via Annexin-V binding, and membrane permeability via PI uptake). (**b**–**k**) Male and female, age- and sex-matched WT (black dots) and *Casp2*^−/−^ (blue squares) BMMs treated with the indicated stimulus for the indicated time. Apoptosis was measured by Annexin-V binding and flow cytometry. Each data point represents one well, with 10,000 events collected per well. Line represents mean ± standard deviation. Data are combined from: (**b**,**c**,**e**,**i**) four experiments each, (**d**,**g**,**h**,**k**) three experiments each, or (**f**,**j**) two experiments each. (**b**) BMMs were treated with TNF (50 ng/mL) and CHX (10 µg/mL): (**b**) Mann–Whitney or (**c**–**k**) two-way ANOVA (for multiple comparisons to assess genotype and dose effects). Comparisons between WT and *Casp2*^−/−^ were not significant (*p* > 0.05) except where indicated. (**c**) * *p* = 0.0385, (**e**) *** *p* = 0.0002.

Though a variety of assays and readouts have been used in the literature [16], we chose to assess apoptosis using flow cytometry and Annexin-V/propidium iodide (PI) staining (Figure 3a). It is well established that the exposure of phosphatidylserine on the outer leaflet of the cell membrane is a reliable readout for apoptosis, with membrane permeability to PI being a good readout for late apoptosis or secondary necrosis [52,53,54]. It is important to note that while some groups excluded debris from their analysis, we chose to include debris particles that likely represent the normal formation of apoptotic bodies during apoptosis (Figure 3a). Indeed, the forward scatter (FSC) and side scatter (SSC) profiles changed depending on the treatment (Figure S2a,b), and smaller debris particles stained positive for Annexin-V (Figure S2c). We chose to include both PI^−^ and PI^+^ events in our analysis to capture all the cells that were undergoing apoptosis, whether it was early or late, respectively [53]. Although our DMSO or PBS control groups showed some baseline levels of cell death, likely due to senescence or the processing of the cells, this did not exceed 10% in most experiments.

To determine whether caspase-2 is involved in the apoptosis of BMMs, we treated wildtype (WT) and *Casp2*^−/−^ cells with published stimuli at various doses and assessed apoptosis. We classified these stimuli based on their cellular effect: death receptor, DNA damage, cytoskeletal disruption, oxidative stress, or heat shock, and we also expanded our search with stimuli utilized in the ferroptosis field (Table 1, inspired by [16]). As a positive control, we used TNF/cycloheximide (CHX) to initiate caspase-8-dependent apoptosis. Though previous studies suggested a role for caspase-2 in this pathway, there was no significant difference between WT and *Casp2*^−/−^ BMMs (Figure 3b).

### 3.4. DNA Damage and Cytoskeletal Disruption

Etoposide, a DNA-damaging agent, inhibits topoisomerase II, causes cell cycle arrest, and eventually causes apoptosis. Etoposide has been extensively studied in the context of caspase-2 [26,55,58,59,60,61,62,63,64,65,66,67,68], but with varying results. Although there may be a caspase-2-dependent effect in Jurkat T-lymphocytes, this effect has not been observed in MEFs or in various human cancer cell lines. We found a sex difference in the response to etoposide, with a greater percent of apoptosis in male BMMs. Although there appeared to be a difference between WT and *Casp2*^−/−^ male BMMs at 10 µM, the loss of caspase-2 did not fully protect against cell death (Figure 3c). Taxol (also called paclitaxel) is another anticancer agent that instead functions by stabilizing microtubules. By inhibiting depolymerization of microtubules, Taxol also causes cell cycle arrest and, eventually, apoptosis. Although HeLa cells showed only a weak cell death phenotype associated with caspase-2 [26], a few groups saw caspase-2-dependent phenotypes in MEFs or splenocytes when comparing WT to *Casp2*^−/−^ [55,61,67,69]. We found no caspase-2-dependent effect in BMMs (Figure 3d).

### 3.5. Oxidative Stress

Based on our hypothesis that the disulfide bond is important during caspase-2 activation, we chose several different stimuli that induce oxidative damage. DHEA, a naturally occurring hormone that can inhibit the pentose phosphate pathway and thus inhibit NADPH production [75], could lead to a redox imbalance that could result in disulfide bond formation in the cytosol. DHEA has been previously shown to induce caspase-2 dimerization in overexpressing HeLa cells [26], and robustly induced cell death in a *Xenopus* system [70], although the involvement of caspase-2 was not knockout validated. After twenty-four hours, 200 µM of DHEA induced cell death in WT BMMs, with slightly better viability in *Casp2*^−/−^ BMMs (Figure 3e), although the loss of caspase-2 did not result in full protection against cell death. Importantly, although DHEA is a sex hormone, we observed similar cell death in BMMs derived from both male and female mice (Figure S3e). Another naturally occurring compound that induces oxidative damage is rotenone, which inhibits complex 1 of the mitochondrial respiratory chain [76]. By inhibiting electron transfer at complex 1, rotenone induces the formation of reactive oxygen species (ROS). It has also been reported that rotenone can bind to tubulin, disrupting microtubule assembly [77]. The role of caspase-2 in rotenone-induced apoptosis has been studied in MEFs [71] and osteoclasts [72]. We saw that *Casp2*^−/−^ BMMs died similarly to WT cells in response to rotenone (Figure 3f). DMNQ, a quinone that functions as a redox cycling agent, has been studied in one report investigating caspase-2 [73]. DMNQ was a good candidate to promote cytosolic disulfide bond formation because it generates superoxide and hydrogen peroxide [78]. However, we did not observe a role for caspase-2 during DMNQ-driven cell death (Figure 3g).

As previously mentioned, SNAP and NONOate are nitric oxide donors that spontaneously release NO in solution [79]. While the role of NONOate in caspase-2-dependent apoptosis has been studied [46,74], the role of SNAP has not been investigated. We did not observe an effect of caspase-2 in cell death after SNAP or NONOate treatment (Figure 3h,i).

### 3.6. Ferroptosis

Ferroptosis is defined by the iron-dependent peroxidation of lipids that culminates in a distinctive form of cell death [80]. A key concept in the ferroptosis field is the excessive formation of radicals and an inability to reduce harmful oxidative species [81,82]. Although ferroptosis was identified over ten years ago, much remains to be discovered about the critical players in this pathway. Importantly, the role of caspase-2 has not been studied in the context of ferroptosis. Because of the imbalance in redox reactions during ferroptosis, we hypothesized that these cellular events may provide the necessary signals for the dimerization of caspase-2 and for the formation of the disulfide bond. Erastin, a small molecule that blocks cystine uptake by inhibiting the system x_c_^−^, diminishes cellular GSH levels and causes ER stress, ultimately leading to ferroptosis [80,83,84]. A related small molecule, RSL3, inhibits glutathione peroxidase 4 (GPX4), causing increased ROS and intracellular iron levels [85,86]. Although both small molecules resulted in BMM death, albeit weakly for erastin, we saw no significant difference between WT and *Casp2*^−/−^ cells (Figure 3j,k). These results suggest that caspase-2 is not involved in ferroptosis of BMMs.

### 3.7. Heat Shock

Several groups have identified a caspase-2-dependent phenotype in the context of heat shock [26,43,55,71]. We saw vastly different amounts of cell death depending on the experimental methods used, with variations depending on the pre-warmed temperature of the media, the position of the cells within the plate, and the number of wells containing cells or media during heat shock. Therefore, we found this method to be difficult to standardize. Nevertheless, in every experiment there was no difference between the WT and *Casp2*^−/−^ cells (Figure S3d).

### 3.8. ROS and In Vivo Infections

Our lab uses in vivo infection models to study programmed cell death pathways. Although several other caspase family members have been well-studied and shown to defend against intracellular infection, fewer studies have investigated the role of caspase-2 during in vivo infection [65,87,88]. During various infections, phagocytes generate ROS to kill intracellular bacteria [89]. Recent studies have shown that ROS can serve additional purposes [90], and we hypothesized that ROS could contribute to redox stress in cells, thus triggering disulfide bond formation in caspase-2. Therefore, we first examined bacterial pathogens against which ROS is a critical defense mechanism.

**Figure 4:**
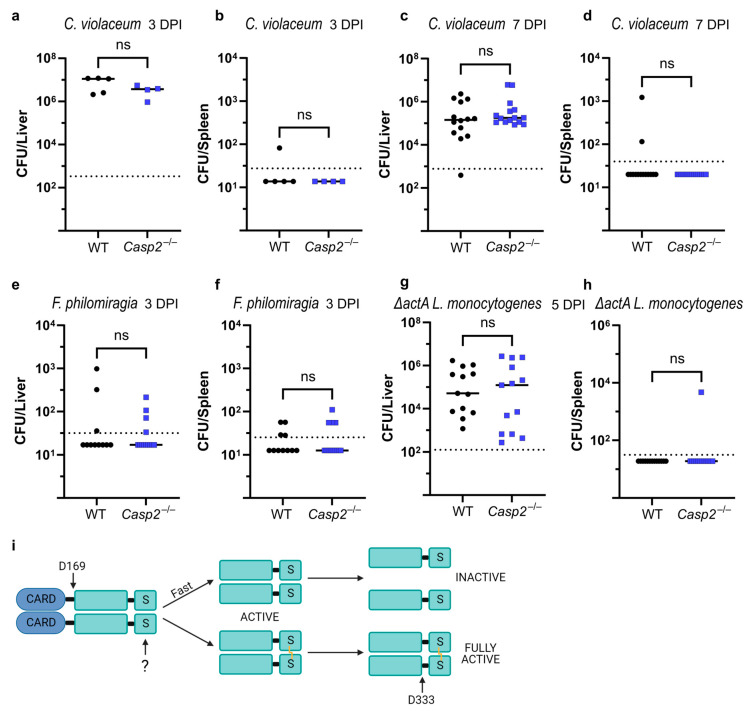
In vivo infection of caspase-2-deficient mice. (**a**–**h**) Livers and spleens from infected WT and *Casp2*^−/−^ mice were harvested at the indicated day post-infection (DPI). Experiments used a mixture of male and female mice, unless otherwise indicated. Each data point represents one mouse (black dots, WT, and blue squares, *Casp2*^−/−^). Line represents the median. Dashed line represents the limit of detection. (**a**,**b**) Intraperitoneal infection with 1 × 10^3^ colony forming units (CFU) *Chromobacterium violaceum*. (**c**,**d**) Intraperitoneal infection with 1 × 10^4^ CFU *Chromobacterium violaceum*. (**e**,**f**) Intraperitoneal infection with 1 × 10^6^ CFU *Francisella philomiragia*. (**g**,**h**) Intravenous infection with 1 × 10^6^ CFU *ΔactA Listeria monocytogenes* using only female mice. (**i**) Hypothesized caspase-2 activation mechanism. Unknown stimulus (?) causes disulfide bond formation. Data are combined from (**a**,**b**) one experiment or (**c**–**h**) two experiments each. (**a**–**f**,**h**) Mann–Whitney test; (**g**) data passed normality test so unpaired two-tailed *t*-test was used; not significant (ns, *p* > 0.05).

*Chromobacterium violaceum* is an environmental bacterium that opportunistically infects immunocompromised individuals, especially those diagnosed with chronic granulomatous disease (CGD) [91]. We, and others, have previously found that mice that lack a functional NADPH oxidase succumb to even low dose infection with *C. violaceum* [92,93]. Interestingly, we also found that *Nos2*^−/−^ mice, which lack a functional inducible nitric oxide synthase, are similarly susceptible, suggesting a nonredundant and protective role for ROS and NO during infection [42]. Because ROS or NO could form the disulfide bond in caspase-2, we hypothesized that caspase-2 might play a role during *C. violaceum* infection. To test this, we infected WT and *Casp2*^−/−^ mice with 1 × 10^4^ CFU, a dose that is lethal to *Nos2*^−/−^ and *Ncf1*^−/−^ mice [42]. At 3 and 7 days post-infection (DPI), we saw no difference in the bacterial burdens between the livers and spleens of WT and *Casp2*^−/−^ mice (Figure 4a–d), indicating that caspase-2 is not essential during infection with *C. violaceum*.

Another opportunistic pathogen that infects CGD patients is *Francisella philomiragia* [94]. Similar to *C. violaceum*, ROS is a key cellular defense mechanism against *F. philomiragia*, and WT mice survive and clear the infection [92]. To test whether caspase-2 is involved in the defense against *F. philomiragia*, we infected WT and *Casp2*^−/−^ mice and assessed burdens at 3 DPI. While the majority of the mice had cleared the infection at this time, a few mice had low burdens in both the liver and spleen, but with no difference between WT and *Casp2*^−/−^ mice (Figure 4e,f). These data indicate that caspase-2 is not required to defend against *F. philomiragia* infection.

Lastly, we hypothesized that *Listeria monocytogenes* could be a good candidate to activate caspase-2 disulfide bond formation. Although *L. monocytogenes* is best known for its ability to cause foodborne illness, several mouse models of infection have been developed [95]. NOS2 is required for resistance to *L. monocytogenes* [96], therefore we hypothesized that the resulting NO stress could activate caspase-2. Although we did confirm the previously published difference between male and female mice [97], we did not see a role for caspase-2 during WT *L. monocytogenes* infection in male mice (Figure S4; note that in this experiment, insufficient female mice were used to make comparisons). Importantly, *L. monocytogenes* encodes several virulence factors that allow it to infect both phagocytic and non-phagocytic cells, and allow it to spread from cell-to-cell via actin-based motility [98]. Specifically in the latter scenario, *L. monocytogenes* uses ActA to polymerize actin and reach neighboring cells. Without ActA, *L. monocytogenes* is still able to infect cells, but is greatly hindered from cell-to-cell spread, resulting in elevated burdens in each infected cell [99]. We hypothesized that the longer dwell time of the bacteria in a single cell could cause redox stress that activates caspase-2. Indeed, *L. monocytogenes* uses glutathione as a trigger to express ActA [100], thus the bacteria could be monitoring for perturbations in the redox state of the host cell. We therefore infected WT and *Casp2*^−/−^ mice with *ΔactA L. monocytogenes*. At 5 DPI, all the mice had detectable liver burdens, but with no difference between WT and *Casp2*^−/−^ mice (Figure 4g,h), suggesting that caspase-2 is dispensable during this infection.

## 4. Discussion

Many questions still remain about the upstream activating signals and the downstream substrates of caspase-2. Indeed, although caspase-2 has been implicated in many phenotypes, the loss of caspase-2 is not completely protective in any of them. Our studies were guided by a simple observation: caspase-2 is uniquely capable of forming a disulfide-bonded dimer. Although this observation was originally made over twenty years ago [21], the disulfide bond has not been the focus of research in the caspase-2 field.

To validate the ability of caspase-2 to form a disulfide-bonded dimer, we used an FKBP domain to induce dimerization of caspase-2 in cells, which allowed us to bypass the uncertainty of upstream activating signals. It was previously shown that the disulfide bond was not required for dimerization [22], but we hypothesized that dimerization is a prerequisite for disulfide bond formation. Indeed, we showed that NO can cause disulfide bond formation of dimerized caspase-2, but disulfide bond formation did not occur in non-dimerized caspase-2. Given this finding, we hypothesize that caspase-2 has a two-step activation mechanism, whereby dimerization occurs first, followed by disulfide bond formation. We hypothesize that complete activation to achieve maximal catalytic activity requires both events. In support of this hypothesis, we saw that inducible dimerization alone was not sufficient to cause cell death in the vast majority of cells.

In further support of the importance of the disulfide bond, Baliga et al. observed that the C436G disulfide bond mutant had roughly half the apoptotic activity of WT caspase-2 when overexpressed [22]. Furthermore, they showed that the purified C436G protein retained only 25% of the catalytic activity of the purified WT protein [22]. The residual activity of the C436 mutant caspase-2 could be explained if the disulfide bond becomes dispensable only when the protein is overexpressed in cells or studied at high concentrations biochemically. In agreement with these observations, the mutation of C436 did not prevent dimerization or cleavage in our inducible dimerization system.

Several factors affect the propensity of cytosolic cysteine residues to form disulfide bonds. These sensitive residues, sometimes called thiol switches [101,102], are influenced by their proximity to the N- or C-terminus, the relative pH of the surrounding solution, and the presence of nearby cysteine or histidine residues, which can all affect the pKa of the thiol in the cysteine [27]. Disulfide bonds can alter cytosolic proteins in multiple ways, by increasing the structural stability, altering protein localization, and enhancing enzymatic activity [24]. As a result, redox-regulated proteins are involved in processes such as the defense against oxidant stress, the prevention of stress-induced protein aggregation, the upregulation of antioxidant metabolic enzymes, or the initiation of signal transduction pathways [24]. Interestingly, the C436 of caspase-2 meets several of the aforementioned criteria, supporting its sensitivity to forming a disulfide bond. Though C436 is in the middle of the dimer interface, it is located within the small subunit of caspase-2 at the C-terminal end, which often enhances cysteine reactivity. Additionally, the crystal structure also revealed that there are several other cysteine residues and histidine residues clustered near C436 (Figure S1c). These nearby residues could facilitate disulfide bond formation by lowering the relative pKa of C436, thereby stabilizing it for nucleophilic attack [27].

There are several cellular contexts in which caspase-2 has been implicated [103], which appear disparate and unrelated. We hypothesize that caspase-2 senses the intracellular redox status, and that the redox status of the cytosol is perturbed during these numerous cellular contexts, such as DNA damage, cytoskeletal disruption, and oxidative stress. Although caspase-2 is activated during these stimuli, we propose that these reflect inappropriate caspase-2 activation, rather than the evolved function of caspase-2. Upon discovering the disulfide-bonded dimer, Schweizer et al. hypothesized that this bond stabilizes the caspase-2 dimer in solution [21], therefore serving a structural role. Furthermore, we hypothesize that disulfide bond formation could be critical for caspase-2 enzymatic activity if it stabilizes the fully active conformation of the dimer. In addition, we hypothesize that the amino acids surrounding C436 might facilitate its reactivity, whereas the catalytic C320 would be unaffected. This is important because high levels of RNS or ROS have been shown to inhibit caspases by inhibiting their enzymatic activity [104]; other cell death initiators can be similarly inhibited [105]. By providing a microenvironment that sensitizes C436 first, we hypothesize that caspase-2 initiates apoptosis before complete loss of the cellular redox state. Lastly, it is also possible that the disulfide bond changes the cellular localization of caspase-2, as there have been reports of caspase-2 localization at the mitochondria [106], ER [107], nucleus [108,109], and Golgi complex [110], although it is unclear whether these are physiologically relevant during the activation of caspase-2. Conversely, the formation of the disulfide bond may only occur at specific cellular localizations because the half-life of ROS and RNS are short, and these molecules preferentially react with nearby targets [32] (although some RNS can travel further [111]). For example, if ROS arises from mitochondria, it will react with molecules nearby, so the activation of caspase-2 may involve its proximity to such sites of oxidative damage.

Several other observations also support our hypotheses. Caspase-2 requires dimerization for activity, and autoproteolytic cleavage at residue D333 (between the large and small subunits) further enhances catalytic activity [22]. We propose that this is actually the second cleavage event. After dimerization, caspase-2 acquires modest enzymatic activity, which we hypothesize will first cleave at D169, between the CARD and the protease domain. This hypothesis is supported by our observation of the C-terminal FLAG tagged caspase-2 construct, which reveals the p34 band generated in the absence of induced dimerization. If caspase-2 is not disulfide bonded, we hypothesize that this D169 cleavage will result in the dissociation of the dimer and the loss of catalytic activity. This hypothesis is inspired by a similar mechanism for caspase-1, which cleaves itself between its CARD and protease domain, releasing the protease dimer from the CARD-inflammasome complex. The released protease then dissociates in a self-limiting mechanism [112]. In support of this hypothesis, we were only able to visualize the disulfide bond using our C320A catalytic cysteine mutant constructs (which are unable to undergo autoproteolytic cleavage at D169), thus maintaining the dimer interface long enough for SNAP or NONOate to form the disulfide bond. Conversely, we hypothesize that if the disulfide bond forms before cleavage at D169, then the dimer is stabilized. The modest catalytic activity will persist, allowing subsequent cleavage at D333 to achieve full activation of caspase-2 (Figure 4i). Furthermore, because the disulfide bond is buried at the center of the dimer, it should be shielded from cytosolic mechanisms that reduce disulfide bonds [21]. We propose that a cellular perturbation that truly dimerizes and disulfide bonds caspase-2 will result in decisive commitment to cell death.

Interestingly, A549 cells were able to express our caspase-2 constructs at higher levels compared to other cell types. In support of this observation, many phenotypes in the caspase-2 field seem to be cell-type specific. We hypothesize that various cell types have different sensitivities to caspase-2 because of differences in the reduction potential and intracellular GSH pools. Indeed, intracellular GSH concentrations can range anywhere from 0.5 to 10 mM depending on the cell type [35], and the availability of GSH can be impacted during various cellular processes, including mitosis [113,114]. Furthermore, caspase-2 has been most extensively studied in immortalized cell lines, which presents additional caveats. Because caspase-2 is specifically inhibited during mitosis [50], the study of caspase-2 in rapidly dividing immortalized cells could lose physiological relevance. Indeed, caspase-2 has been implicated in processes that could all occur during aberrant mitosis (i.e., DNA damage, cytoskeletal disruption, oxidative stress, etc.), and mitotic processes are frequently altered in cancerous cell types. Furthermore, immortalized cells often have abnormal expression of several cell cycle related proteins, such as pRb [115] and p53 [116], the latter being linked to caspase-2 in multiple contexts, especially in relation to PIDD1 [10,44,117]. Lastly, mitosis is an energetically taxing process that causes increased levels of ROS [118] and elevated pH [119]. In extreme cases, metabolic stress compromises antioxidant levels, such as GSH [120,121]. Importantly, caspase-2 has also been implicated in metabolic homeostasis [122,123,124]. Therefore, mitosis could cause caspase-2 disulfide bond formation via a myriad of mechanisms. We hypothesize that caspase-2 is inhibited during mitosis because events occur during proliferation that would erroneously activate caspase-2. Indeed, other cell death proteins such as NLRP3 are inhibited during mitosis, likely due to the same risk of autoactivation [125].

To test the requirement of the disulfide bond in caspase-2-dependent cell death phenotypes, we generated C436A point mutant mice, which will allow us to generate *Casp2*^C436A/C436A^ mutant BMMs and MEFs (unpublished). Unfortunately, we were not able to identify a caspase-2-dependent cell death phenotype with which to test our hypotheses, further emphasizing that phenotypes in the literature are often cell-type specific. It is possible that BMMs, a cell type adept at producing ROS and RNS [126], are protected from many proposed stimuli. Although neutrophils and macrophages cooperate to kill bacteria [127], there could be a context in which too much ROS and RNS could compromise cells. Once a cell reaches the point of no return (i.e., intracellular GSH levels cannot be recovered), apoptosis may be the best outcome to prevent tissue damage. Standard tissue culture techniques also raise a concern because cells are exposed to 21% oxygen and, thus, have adapted to oxidative stress. To expand our search for a phenotype, we also generated *Casp2*^−/−^ MEFs. However, we were unable to use the Annexin-V/PI assay with this cell type because simply lifting the cells caused a high percentage of Annexin-V positivity, which we were unable to eliminate despite numerous efforts to optimize the lifting protocol.

## 5. Conclusions

If our hypothesis is correct, several questions still remain. What is the evolved function of caspase-2? During what cellular context does the disulfide bond form as a defense mechanism to initiate apoptosis? Because the maintenance of redox homeostasis is a basic function of every cell, the number of possible routes to cause caspase-2 activation are numerous. Caspase-2 should only respond during an extreme redox stress event, because it is not required during normal redox homeostasis. *Casp2*^−/−^ mice are viable and healthy, unlike *Keap1*^−/−^ mice, whose role in normal redox homeostasis is essential to life [128]. Instead, we hypothesize that caspase-2 senses extreme perturbation in redox homeostasis that occurs after insults that have yet to be identified. Because many other caspase family members have been implicated during the immune response to infection, perhaps the evolved function of caspase-2 is also to protect cells from intracellular infection. Though cytosolic infection is a feat not accomplished by many bacteria, some pathogens have evolved virulence strategies that allow them to replicate in the cytosol [98], utilizing host cell nutrients for their own gain. In this context, capsase-2 could sense the metabolic strain caused by cytosolic bacterial or viral replication. However, known host-adapted pathogens are likely to avoid causing this degree of stress, or they may directly inhibit caspase-2. Notwithstanding all of these potential connections, the fields of metabolism, redox homeostasis, mitosis, apoptosis, and infection are all complicated in their own regard [129,130]; the study of them altogether, in the context of caspase-2, will surely be a monumental task.

## Figures and Tables

**Table 1:** Summary of selected stimuli studied in the caspase-2 field.

Stimulus	Previously Tested Cell Type	Cellular Effect	References
TNF/CHX	HeLa; Jurkat; MEF; IMR90E1A; MCF7	Death receptor	[26,55,56,57]
Etoposide	Jurkat; MEF *; IMR90E1A; HeLa; HCT116; BMM *; U2OS *; U937	DNA damage	[26,55,58,59,60,61,62,63,64,65,66,67,68]
Taxol	HeLa; Jurkat; MEF *; Splenocytes *	Cytoskeletal disruption	[26,55,61,67,69]
DHEA	HeLa; *Xenopus*	Oxidative stress	[26,70]
Rotenone	MEF *; osteoclasts *	Oxidative stress	[71,72]
DMNQ	Neural stem cells	Oxidative stress	[73]
Heat shock	HeLa; Jurkat; Splenocytes * +; MEF *	Heat shock	[26,43,55,71]
NONOate	3T3; H4; BMK	Oxidative stress	[46,74]
SNAP	―	Oxidative stress	This work
Erastin	―	Ferroptosis	This work
RSL3	―	Ferroptosis	This work

* Indicates *Casp2*^−/−^ cells; + indicates stimulation with 12-O-tetradecanoylphorbol-13-acetate (TPA) (50 ng/mL) and ionomycin (1 µg/mL).

## Data Availability

The data presented in this study are available on request from the corresponding author (Duke privacy policy). Vector maps of novel constructs have been saved in SnapGene (San Diego, CA, USA) and will be made available upon request.

## Supplementary Materials

The following supporting information can be downloaded at: https://www.mdpi.com/article/10.3390/biology13010049/s1, Figure S1: Cystal structure of dimerized caspase-2 and inducible dimerization system; Figure S2: Visualization of cell death caused by inducible dimerization of caspase-2; Figure S3: Quantification of apoptosis by Annexin-V/PI and flow cytometry; Figure S4: Infection of *Casp2*^−/−^ mice with WT *Listeria monocytogenes*; Figure S5: Original Western blots corresponding to Figure 1; Figure S6: Original Western blots corresponding to Figure 2, Materials and Reagents. Reference [131] is cited in the supplementary materials.
