# Supplementary material for: Validation of the Intermolecular Disulfide Bond in Caspase-2
Source: Biology (Basel). 2024 Jan 17;13(1):49. doi: 10.3390/biology13010049 (PMC10813798; doi:10.3390/biology13010049)
Supplement: Supplementary file 1 [file biology-13-00049-s001.zip › biology-2796408-supplementary.pdf]

Supplementary Materials

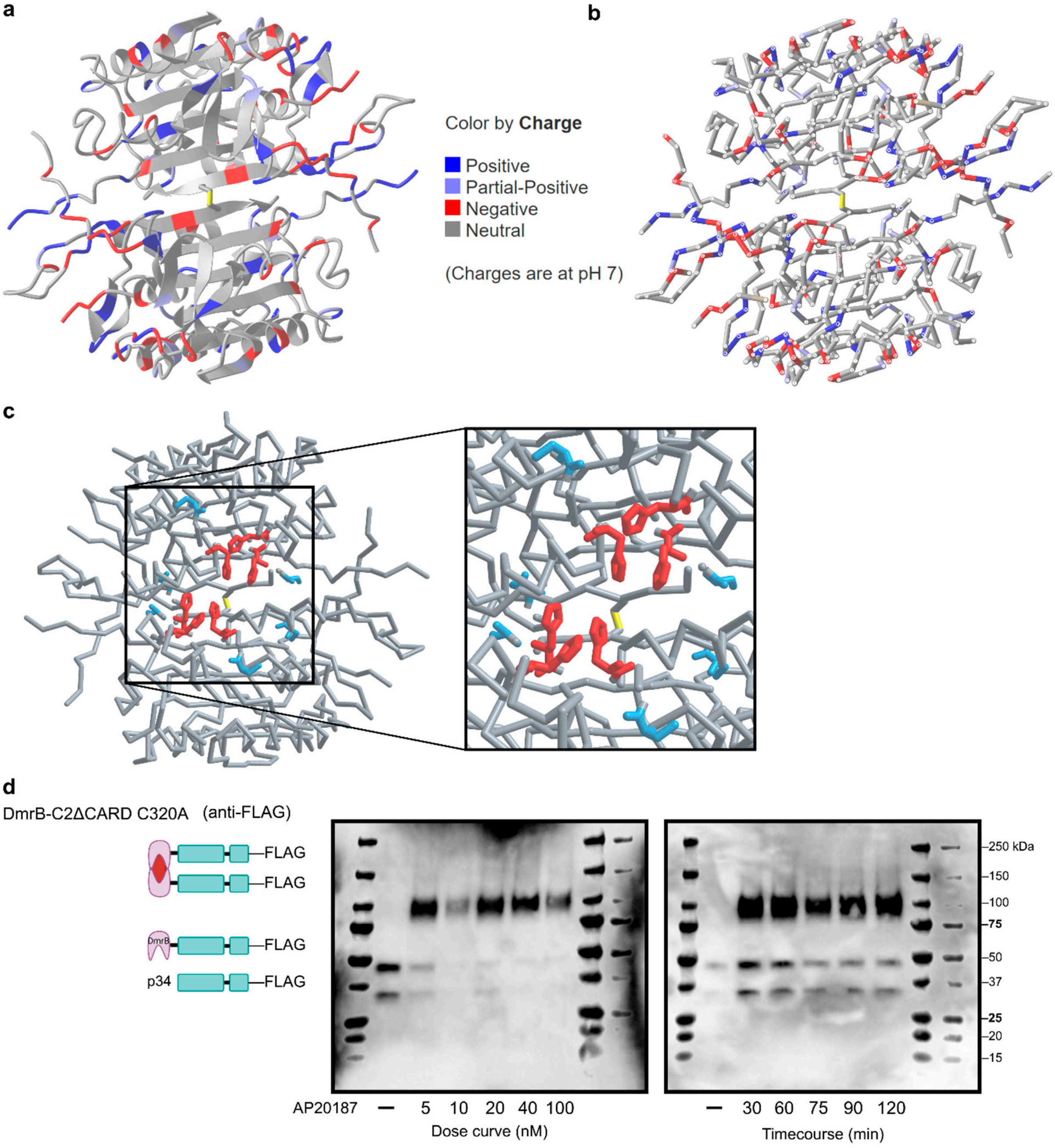

Figure S1. Crystal structure of dimerized caspase-2 and inducible dimerization system. (a–c) Alternative visualization methods for the crystal structure of dimerized caspase-2 published in [21]. Disulfide bond highlighted yellow. **(a–b)** Structure colored by charge. **(c)** Structure colored gray, with selected histidine residues colored red (H187, H402, and H442) and cysteine residues colored blue (C366, C399 and C440). Each colored residue appears twice, once per monomer. **(d)** Western blot analysis of whole cell lysates from transduced A549 cells stably expressing DmrB-C2ΔCARD C320A. Prior to lysis, cells were treated with indicated concentration of AP20187 for 2 hours, or for indicated time with 40 nM of AP20187. Lysates were then treated with DSS crosslinker for 30 minutes.

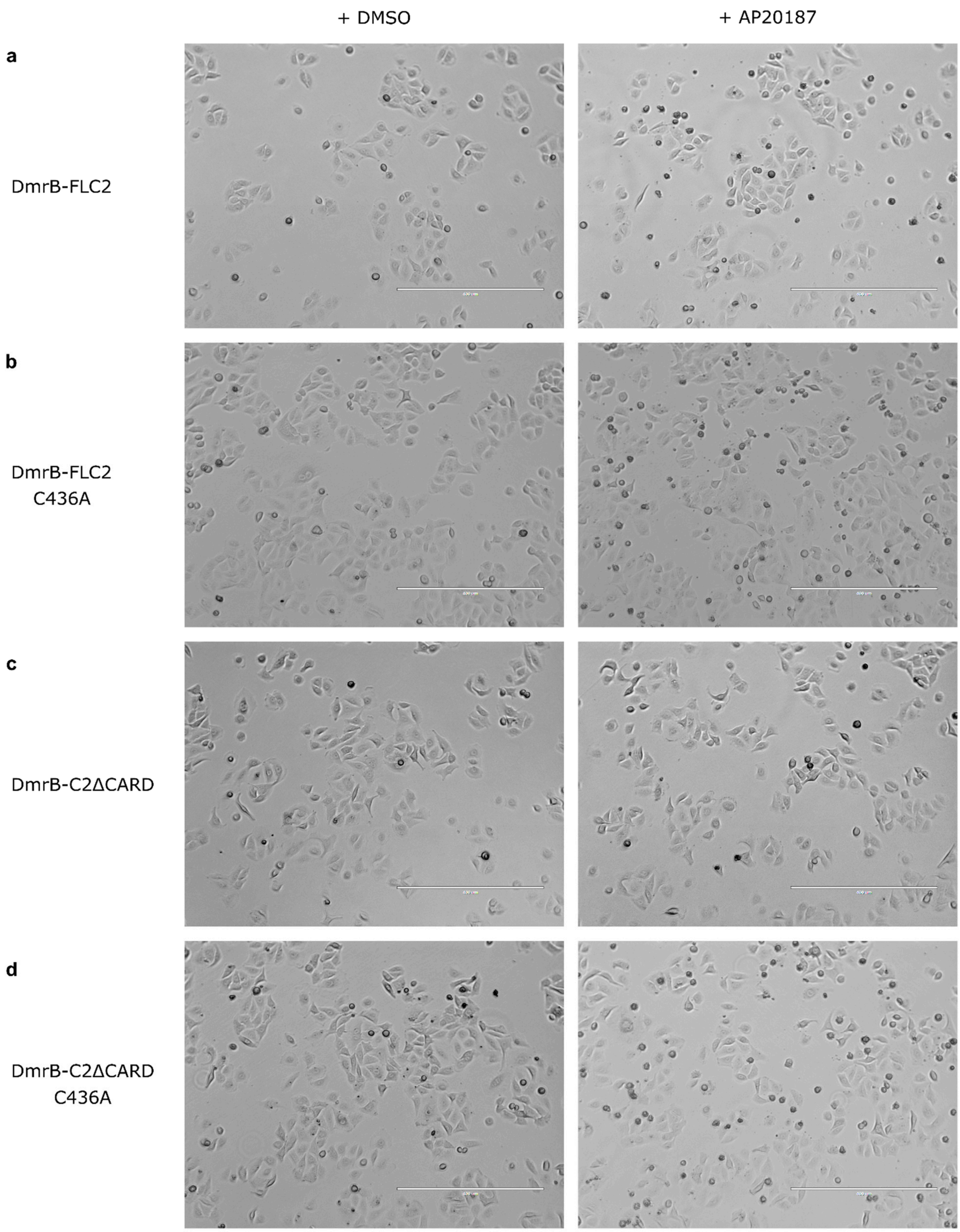

Figure S2. Visualization of cell death caused by inducible dimerization of caspase-2. Transduced A549 cells stably expressing DmrB constructs. Microscopic brightfield images were taken after treatment with DMSO (control) or AP20187 for 4 hours.

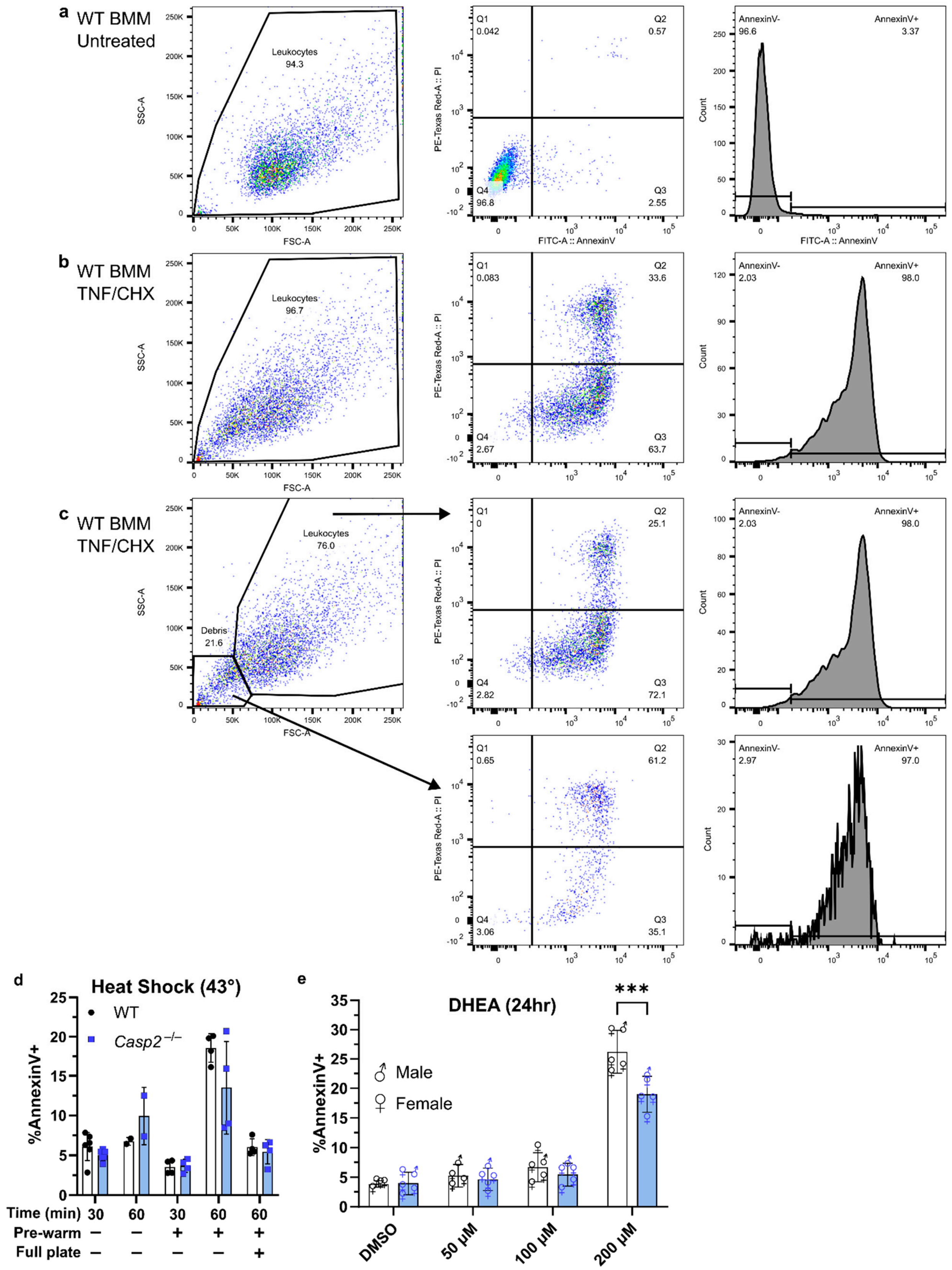

Figure S3. Quantification of apoptosis by Annexin-V/PI and flow cytometry. (a) Untreated WT BMMs. Gating strategy: Leukocytes → Annexin-V Histogram. Both PI^- and PI^+ cells were included in the Annexin-V^+ population. **(b)** WT BMMs treated with TNF (50 ng/mL) and CHX (10 μg/mL) for 4 hours. Forward scatter (FSC) vs side scatter (SSC) profile changes with treatment. The majority (98%) of cells stain positive for Annexin-V, with approximately 34% staining double positive (Annexin-V^+PI^+). **(c)** Same sample as in (b), but alternatively gated to exclude small debris particles from

leukocyte population. Debris particles stain positive for Annexin-V, with approximately 61% staining double positive (Annexin-V$^+$PI$^+$). **(d-e)** Male and female, age- and sex-matched WT and *Casp2*$^{-/-}$ BMMs treated with the indicated stimulus for the indicated time. Apoptosis was measured by Annexin-V binding and flow cytometry. Each data point represents one well, with 10,000 events collected per well. Line represents mean ± standard deviation. (d) For heat shock treatment of cells, plates containing cells cultured at 37° were placed in an incubator warmed to 43° for the indicated amount of time. In some experiments, media was first replaced with cDMEM that was pre-warmed to 43°. Cells were then returned to a 37° incubator to recover for 24 hours. Data are combined from four experiments. (e) Same data as in Figure 3e, but with male and female samples annotated. Though DHEA is a sex hormone, we found no marked difference between male and female cells in the first experiment. In three additional experiments, we used what cells we happened to have stored in liquid nitrogen, which happened to be female. We did not perform both sexes in all replicates because it is experimentally cumbersome to double the number of samples, as we did in the first experiment. Data are combined from four experiments. Two-way ANOVA (for multiple comparisons to assess genotype and dose effects). Comparisons between WT and *Casp2*$^{-/-}$ were not significant (p>0.05) except where indicated. ***p = 0.0002.

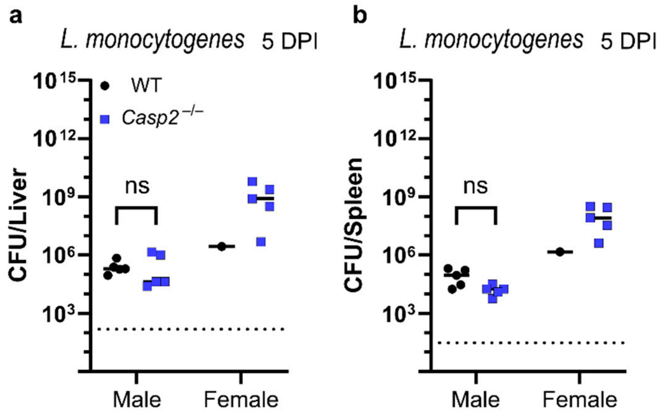

Figure S4. Infection of *Casp2*$^{-/-}$ mice with WT *Listeria monocytogenes*. Intravenous infection of WT and *Casp2*$^{-/-}$ mice with 1x10^4 CFU *Listeria monocytogenes*. Livers and spleens were harvested at the indicated day post-infection (DPI). Each data point represents one mouse. Line represents median. Dashed line represents limit of detection. One WT female mouse succumbed to infection prior to harvest; data are from one experiment. Due to small sample size for females, statistical analysis was only performed on male samples; unpaired two-tailed t-test, not significant (ns, p>0.05).

a (corresponds to Figure 1c)

(anti-Casp2)

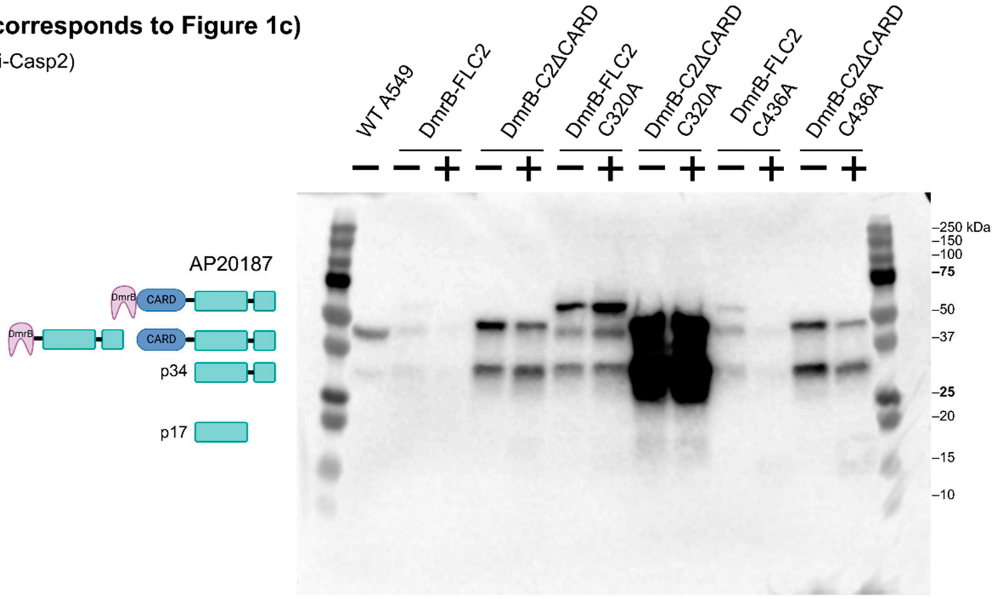

b (corresponds to Figure 1d)

(anti-Casp2)

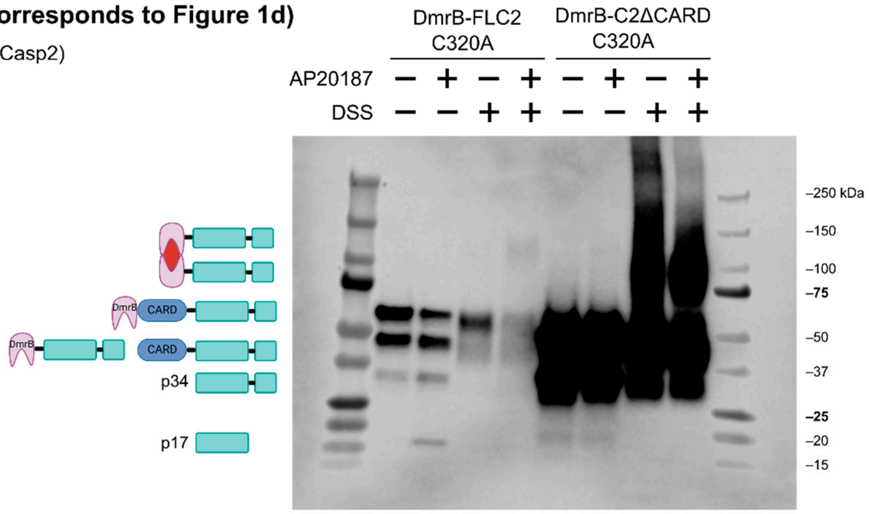

Figure S5. Original western blots corresponding to Figure 1.

a (corresponds to Figure 2b)

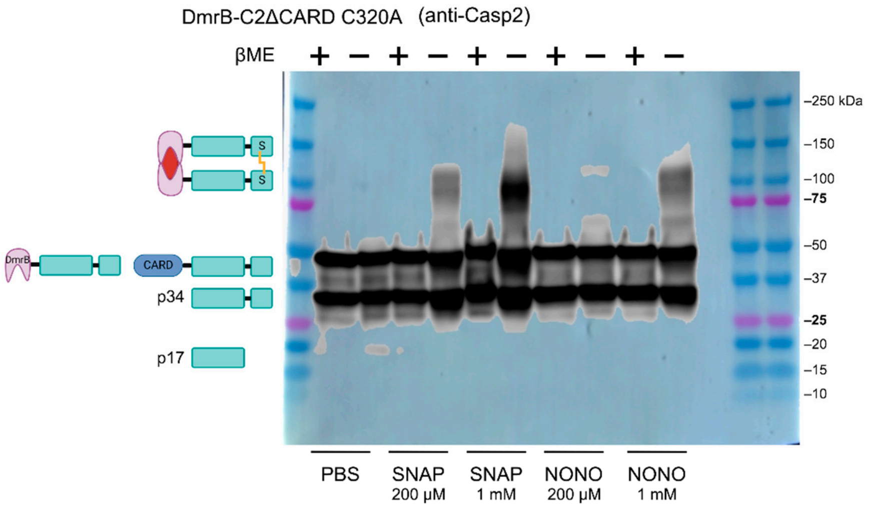

b (corresponds to Figure 2c)

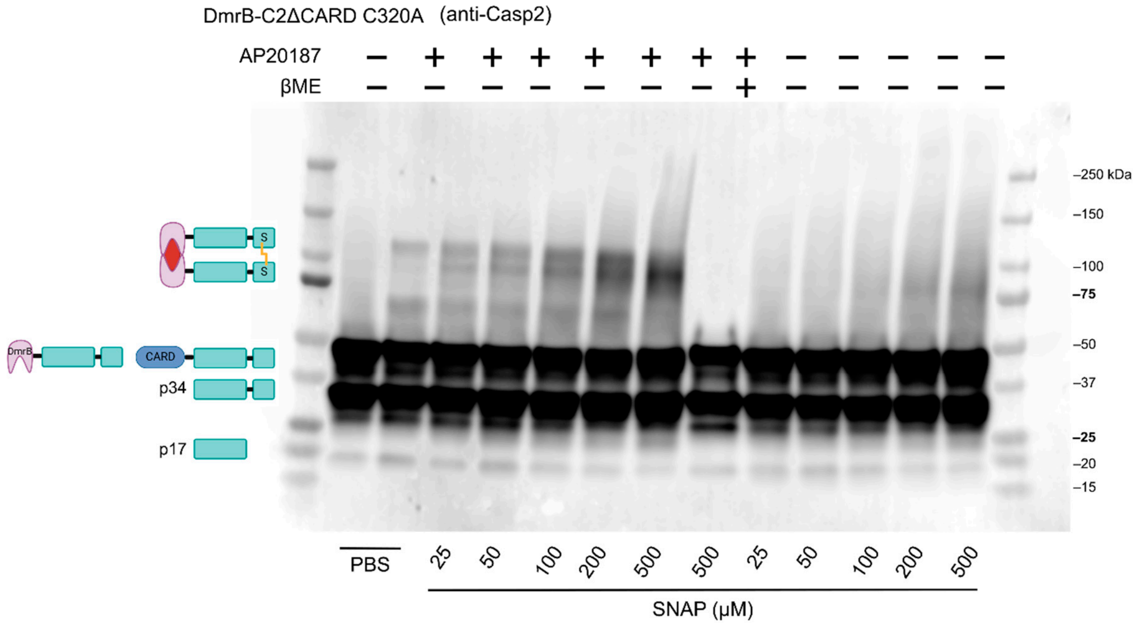

Figure S6. Original western blots corresponding to Figure 2.

Materials and Reagents

Mice

- *Casp2*$^{-/-}$ mice (Reference [17] cited in main text; Jackson Laboratory Ref# 007899; gift from Anna Mae Diehl)
- Wildtype C57BL/6 mice (Jackson Laboratory; Ref# 000664)

Cell Lines

- Phoenix-AMPHO (ATCC Ref# CRL-3213; gift from Alan Aderem)
- L-929 Cells (Duke Cell Culture Facility, CCL-1, NCTC clone 929)
- A549 human lung epithelial cells (ATCC Ref# CCL-185; gift from William E. Goldman)

Vectors

- pHom-1 homodimer plasmid (pC4-Fv1E originally from Ariad, now available through Clontech Laboratories, Inc. as the iDimerize™ Inducible Homodimer System; Ref# 635068)
- pMXs-IP or pMXs-IP-eGFP (Reference [131] cited in Supplementary Materials; gift from Alan Aderem)

Genotyping and Cloning

- Agarose (Thermo Scientific™; Ref# BP1356)
- 50x TAE Buffer (Bio-Rad; Ref# 1610773)
- Labnet International Enduro™ Power Supplies 300V
- Molecular Biology Grade Water (Corning™; Ref# 46-000-CM)
- SYBR™ Safe DNA Gel Stain (Invitrogen™; Ref# S33102)
- GeneRuler 1 kb Plus DNA Ladder (Thermo Scientific™; Ref# SM1331)
- Q5® High-Fidelity DNA Polymerase (New England Biolabs Inc.; Ref# M0491L)
- SpeI-High-Fidelity (HF®)(New England Biolabs Inc.; Ref# R3133S)
- PacI (New England Biolabs Inc.; Ref# R0547S)
- NotI-High-Fidelity (HF®)(New England Biolabs Inc.; Ref# R3189S)
- KAPA HotStart Mouse Genotyping Kit (Roche; Ref# 07961804001)
- Jax Protocol 29310 (WT vs *Casp2*$^{-/-}$ found at https://www.jax.org/Protocol?stockNumber=007899&protocolID=29310):
    - Primer 1: CTC ACT GGC TAC CTA ACT TCC
    - Primer 2: CCA TGC ATT GGG AGA CAC TTA C
    - Primer 3: TTG GCG CTA CCG GTG GAT GTG GAA TGT G

Transfection and Transduction

- Lipofectamine™ 3000 Transfection Reagent (Invitrogen™; Ref# L3000001)
- Opti-MEM™ Reduced Serum Medium, no phenol red (Gibco™; Ref# 11058021)
- 0.45 µm Syringe Filter (Genesee Scientific; Ref# 25-246)
- Polybrene Transfection Reagent (Millipore™; Ref# TR-1003-G)

Cell Culture

- Thermo Scientific™ Heracell™ 150i CO_2 Incubator
- Eppendorf® centrifuge (model 5810 R)
- Beckman Coulter Allegra® X-15R Centrifuge
- Invitrogen™ EVOS™ Inverted Fluorescent Microscope
- 1X DMEM, +4.5 g/L D-Glucose, +L-Glutamine, +110 mg/L Sodium Pyruvate (Gibco™; Ref# 11995-065)
- PenStrep +10,000 Units/mL Penicillin, +10,000 µg/mL Streptomycin (Gibco™; Ref# 15140-122)
- HyClone™ Characterized Fetal Bovine Serum (Cytiva; Ref# SH30396.03)
- 1X DPBS, -Calcium Chloride, -Magnesium Chloride (Gibco™; Ref# 14190-144)
- Trypsin-EDTA 0.25% (Gibco™; Ref# 25200056)
- 70 µm Cell Strainers (Genesee Scientific; Ref# 25-376)
- 24-well plates, Non-TC treated (Genesee Scientific; Ref# 25-102)
- 24-well plates, TC-treated (Greiner Bio-One; Ref# 662 160)
- 6-well plates, Non-TC treated (Genesee Scientific; Ref# 25-100)
- 6-well plates, TC-treated (Genesee Scientific; Ref# 25-105)

- 15-cm dishes, Non-TC treated (Genesee Scientific; Ref# 32-106)
- 10-cm dishes, Non-TC treated (Fisherbrand™; Ref# FB0875711Z)
- Mr. Frosty™ Freezing Container (Thermo Scientific™; Ref#5100-0001)
- Freezing media (10% DMSO (Fisher Chemical™; Ref# D128-500) in FBS)
- 1.8 mL Self-Standing Cryovials (Genesee Scientific; Ref# 24-202P)
- 30G Needles (Exel International; Ref# 26437)
- 23G Needles (Exel International; Ref# 26407)
- 1 mL Syringes (Fisherbrand™; Ref# 14955462)

SDS-PAGE and Western Blot
- BioTek® Plate Reader
- Azure Biosystems 500® imager
- IGEPAL® CA-630 (Sigma-Aldrich; Ref# I8896)
- BCA Protein Assay Kit (Pierce™; Ref# 23225)
- Laemmli Sample Buffer (Bio-Rad; Ref#1610747)
- 2-Mercaptoethanol (βME)(Sigma-Aldrich; Ref# M3148)
- Dual Color Standard (Bio-Rad; Ref# 1610374)
- All Blue Standard (Bio-Rad; Ref# 1610373)
- 8-16% Mini-PROTEAN® TGX™ Precast Protein Gels (Bio-Rad; Ref#4561103)
- 4-15% Mini-PROTEAN® TGX™ Precast Protein Gels (Bio-Rad; Ref# 4561086)
- Mini-PROTEAN® Tetra Cell System (Bio-Rad; Ref# 1658004EDU)
- Pierce™ ECL Western Blotting Substrate (Thermo Scientific™; Ref# 32106)
- Immobilon®-P Transfer Membrane (Millipore™; Ref# IPVH85R)

Antibodies
- Rat anti-Caspase 2 antibody, clone 10C6 (Sigma-Aldrich, MAB3501)
- Mouse anti-FLAG® antibody, clone M2 (Sigma-Aldrich, F3165)
- Peroxidase AffiniPure Goat anti-rat, polyclonal (Jackson ImmunoResearch, Ref# 112-035-062)
- Peroxidase AffiniPure Goat anti-mouse, polyclonal (Jackson ImmunoResearch, Ref# 115-035-062)

Stimuli/Drugs
- AP20187 (Sigma-Aldrich; Ref# SML2838)
- DSS (disuccinimidyl suberate) (Thermo Scientific™; Ref# 21655)
- SNAP (Sigma-Aldrich; Ref# N3398)
- NONOate (Cayman Chemical; Ref# 82100)
- Recombinant murine TNF-alpha (PeproTech; Ref# 315-01A)
- Cycloheximide (Tocris; Ref# 0970)
- Etoposide (Sigma-Aldrich; Ref# E1383)
- Paclitaxel (Taxol) (Invitrogen™; Ref# P3456)
- Dehydroepiandrosterone (DHEA) (Cayman Chemical; Ref# 15728)
- Rotenone (Sigma-Aldrich; Ref# R8875)
- DMNQ (Cayman Chemical; Ref# 19571)
- Erastin (MedChemExpress; Ref# HY-15763)
- RSL3 (Cayman Chemical; Ref# 19288)

Flow Cytometry
- Dead Cell Apoptosis Kit, Alexa Fluor™ 488, Propidium Iodide (Invitrogen™; Ref# V13245)
- Falcon™ Round-Bottom Polystyrene Test Tubes (Thermo Scientific™; Ref# 14-959-1A)
- BD LSRFortessa™ X-20 Cell Analyzer (Duke Flow Cytometry Core Facility)

Bacteria
- *Chromobacterium violaceum* (ATCC Ref# 12472)
- *Francisella philomiragia* (CGD isolate, gift from Stephen Holland)
- *Listeria monocytogenes* (10403s; gift from Daniel A. Portnoy)
- *ΔactA Listeria monocytogenes* (gift from Daniel A. Portnoy)

Bacterial Culture and Tissue Harvesting

- Fisherbrand™ Bead Mill 24 Homogenizer (Ref# 15-340-163)
- Retsch® Mixer Mill MM400 Homogenizer
- Agar (Fisher BioReagents™; Ref#BP1423-2)
- Chocolate Agar plates (Hardy Diagnostics; Ref# E14)
- LB Broth (Apex™ BioResearch; Ref#11-120)
- BHI Broth (Millipore™; Ref#53286)
- 7 mL homogenizer tubes (Omni International; Ref# 19-651)
- 1 mL homogenizer tubes (Fisherbrand™; Ref# 14-666-315)
- 5 mm Stainless Steel Beads (QIAGEN; Ref# 69989)
